# Radiographical findings in patients with liver cirrhosis and hepatic encephalopathy

**DOI:** 10.1093/gastro/gov049

**Published:** 2015-10-13

**Authors:** Saleh Elwir, Hassan Hal, Joshua Veith, Ian Schreibman, Zakiyah Kadry, Thomas Riley

**Affiliations:** ^1^Department of Internal Medicine, Penn State Milton S. Hershey Medical Center, Hershey, Pennsylvania (PA), USA; ^2^Department of Radiology, Penn State Milton S. Hershey Medical Center, Hershey, PA, USA; ^3^Penn State Milton S. Hershey Medical Center and School of Medicine, Hershey, PA, USA; ^4^Division of Gastroenterology and Hepatology, Penn State Milton S. Hershey Medical Center, Hershey, PA, USA; ^5^Division of Transplant Surgery, Penn State Milton S. Hershey Medical Center, Hershey PA, USA

**Keywords:** hepatic encephalopathy, cirrhosis, portal vein diameter, computed tomography (CT)

## Abstract

**Background and aims:** Hepatic encephalopathy is a common complication encountered in patients with liver cirrhosis. Hepatic encephalopathy is not reflected in the current liver transplant allocation system. Correlation was sought between hepatic encephalopathy with findings detected on radiographic imaging studies and the patient’s clinical profile.

**Methods:** A retrospective analysis was conducted of patients with cirrhosis, who presented for liver transplant evaluation in 2009 and 2010. Patients with hepatocellular carcinoma, ejection fraction less than 60% and who had a TIPS (transjugular intrahepatic portosystemic shunting) procedure or who did not complete the evaluation were excluded. Statistical analysis was performed and variables found to be significant on univariate analysis (*P <* 0.05) were analysed by a multivariate logistic regression model.

**Results:** A total of 117 patients met the inclusion criteria and were divided into a hepatic encephalopathy group (*n =* 58) and a control group (*n =* 59). Univariate analysis found that a smaller portal vein diameter, smaller liver antero-posterior diameter, liver nodularity and use of diuretics or centrally acting medications showed significant correlation with hepatic encephalopathy. This association was confirmed for smaller portal vein, use of diuretics and centrally acting medications in the multivariate analysis.

**Conclusion:** A decrease in portal vein diameter was associated with increased risk of encephalopathy. Identifying patients with smaller portal vein diameter may warrant screening for encephalopathy by more advanced psychometric testing, and more aggressive control of constipation and other factors that may precipitate encephalopathy.

## Introduction

Cirrhosis is associated with multiple hemodynamic changes that can be assessed by invasive (transjugular pressure measurement) and non-invasive means (liver duplex) [1]. Through the transjugular route, portal venous pressure gradients can be measured and a measurement exceeding 5 mm Hg is diagnostic of portal hypertension [2]. Duplex studies can detect the presence or absence of portal vein thrombosis and assess the diameter, flow rate and direction of portal venous flow [3].

Doppler ultrasound is a valuable non-invasive tool in the evaluation of the hemodynamics of patients with cirrhosis and portal hypertension. Hepatic artery pulsatility index (peak systolic velocity *minus* end diastolic velocity, *divided by* the mean velocity) correlated directly with portal venous pressure gradient [4]. Hepatic venous flow pattern is another parameter that was investigated in patients with cirrhosis. The changes in hepatic venous waveforms correlated with the extent of hepatic fibrosis. Certain waveforms were associated with poorer prognosis and lower five-year survival, even among patients with the same Child-Pugh score [5].

Hepatic encephalopathy (HE) is a frequent complication and one of the most debilitating manifestations of liver disease, severely affecting the lives of patients and their care providers [6]; it is also associated with increased mortality [7]. HE encompasses a broad range of neuropsychiatric deficits, from clinically undetectable abnormalities, which are only apparent on psychometric evaluation, to confusion, coma and death [8]. Although increased levels of serum ammonia are thought to play a key role in the pathophysiology of HE, the diagnosis of this entity remains a clinical process [9]. Psychometric and neurophysiological testing are available and may be used for the evaluation of patients with suspected minimal or covert HE. The tests require experienced professionals to administer them and are time-consuming. Also, none of the available tests are specific for HE, especially if confounding factors—such as neuropsychiatric disorders, psychoactive medication, or current alcohol use—are present [6]. The most recent clinical guidelines by the American Association for the Study of Liver Disease (AASLD) and the European Association for the Study of the Liver (EASL) state that an operational approach may be to test patients who have problems with their quality of life, or in whom there are complaints from the patients and their relatives [6].

In this study we reviewed imaging studies [computed tomography (CT)/magnetic resonance imaging (MRI) and Liver duplex ultrasound.] in patients with liver cirrhosis, who were evaluated for liver transplantation. We planned on assessing whether any of the findings detected on the aforementioned studies had any correlation with HE that could predict or explain the patients' clinical status.

## Patients and methods

In the period between 1 January 2009 and 31 December 2010, 252 patients were evaluated in the liver transplant clinic at Penn State Milton S. Hershey Medical Center. Patients with hepatocellular carcinoma (HCC) (*n =* 52), ejection fraction less than 60% (*n =* 7), those who had a TIPS (transjugular intrahepatic portosystemic shunting) procedure (*n =* 6) and patients who did not complete the evaluation (*n =* 70) were excluded from the analysis; patients with fulminant liver failure were also not included in the analysis. These patients were excluded because HE is a known complication encountered in patients who undergo TIPS procedure and patients with low ejection fraction may have altered hemodynamics that may present in a fashion similar to portal hypertension. Patients with HCC are usually referred to a transplant clinic early in the course of their disease and they frequently have HCC as the only manifestation of their liver disease. Patients with portal vein thrombosis were not excluded from this analysis, in order to assess any contribution it may make to HE.

**Figure 1.**
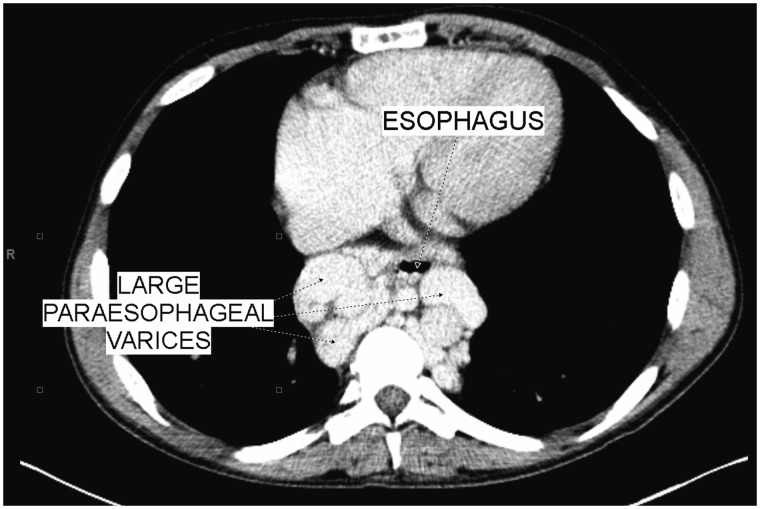
CT scan of the abdomen showing large paraesophageal varices. In this patient, portal vein diameter was measured at 8 mm.

All imaging studies were made within three months of the evaluation date. Liver duplexes, abdominal CT scans and MRIs of these patients were reviewed by one attending radiologist, who was blinded to the clinical information. Liver antero-posterior and cranio-caudal diameters were measured from the right hepatic lobe. The largest spleen diameter (whether antero-posterior or cranio-caudal) was measured. Portal vein diameter was assessed at the *porta hepatis* by CT scan (Figure 1). Given that the timing of the measurement of the portal vein in relation to respiration could not be confirmed in this retrospective study, only measurements of the portal vein by CT/MRI were used. Resistive Index (RI), measured by duplex ultrasonography, was calculated by the following equation:

RI=(peak systolic velocity–end diastolic velocity)÷peak systolic velocity [3].

Paraesophageal, splenorenal varices and umbilical vein recanalization were reported if the CT scan imaging showed any evidence of their presence, regardless of size.

One hundred and seventeen patients were included in the study. They were divided into an HE group (*n =* 58) and a control group (*n =* 59) based on whether or not they exhibited clinical evidence of HE. Patients were included in the HE group if they had a current or previous diagnosis of Grade 2 or higher HE according to the West Haven criteria [10]. The findings of the imaging studies, clinical and laboratory data were compared between the two groups. The study was approved by the Penn State Milton S. Hershey Medical Center Institutional Review Board in September 2011.

## Statistical analysis

Univariate analysis was carried out with chi-squared tests for binomial variables and analysis of variance (ANOVA) for continuous variables. Odds ratios (ORs) and 95% confidence intervals (CIs) were calculated for selected variables that were identified as significant in the univariate analysis. The concordance index (c-index) was also reported. Selected variables were evaluated by a multivariate logistical regression model. A *P*-value of less than 0.05 was considered significant.

## Results

### Patient population

The most common causes of cirrhosis in this cohort were alcohol (18%), hepatitis C virus (HCV) (21%) and a combination of HCV and alcohol (21%). The HE and control groups were comparable in terms of age, sex, race and model for end-stage liver disease (MELD) scores (Table 1).

**Table 1.** Baseline characteristics of the study population

Variable	HE group (*n =* 58)	Control group (*n =* 59)	*P*-value
Age (years)	54.6 ± 8.8	54.3 ± 10.6	>0.05
Female gender	24 (41.4)	16 (27.1)	>0.05
Body mass index	29.9 ± 6.1	28.3 ± 6.2	>0.05
MELD score	13.9 ± 5.0	12.7 ± 4.1	>0.05
Etiology of cirrhosis			>0.05
Alcohol	10 (17%)	11 (19%)	
HCV	16 (28%)	9 (15%)	
Alcohol + HCV	14 (24%)	10 (17%)	
PSC	2 (3%)	9 (15%)	
NASH	9 (16%)	8 (14%)	
Other^a^	7 (12%)	12 (20%)	

Data expressed as mean ± standard deviation or number (%).

HE = hepatic encephalopathy; HCV = hepatitis C virus; MELD = model for end-stage liver disease; NASH = non-alcoholic steatohepatitis; PSC = primary sclerosing cholangitis

^a^Others include 2 PBC (primary biliary cirrhosis), 1 cryptogenic, 1 HBV (hepatitis B virus), 1 hemochromatosis, 1 Wilson, and 1 autoimmune hepatitis in the HE group, and 3 PBC, 3 cryptogenic, 2 HBV, 2 autoimmune hepatitis, 1 hemochromatosis and 1 alpha one antitrypsin deficiency in the control group.

When correlating the patients' historical data and medication profiles to their presentation, patients using centrally acting medications (antidepressants, sleep aids, benzodiazepines or antipsychotics) were more likely to have HE (*P =* 0.002). Patients with HE were more likely than the controls to be on diuretics (*P =* 0.002) (Table 2).

**Table 2.** Correlation of various historical data, laboratory test results and imaging values with HE

Variable	HE group (*n =* 58)	Control group (*n =* 59)	*P*-value
**Historical information**			
Esophageal varices	60%	66%	0.518
Variceal bleeding	21%	24%	0.693
Variceal band ligation	24%	24%	0.952
**Medications**			
Non-selective beta blockers	36%	44%	0.386
Narcotics	14%	8%	0.394
Centrally acting medications^a^	41%	15%	**0.002**
Diuretics^b^	88%	63%	**0.002**
**Laboratory tests**			
Na <135 mmol/L	69%	68%	0.892
Creatinine > 1 mg/dL	31%	29%	0.793
**CT scan findings**			
Liver antero-posterior diameter ≤6 cm	91%	68%	**0.001**
Liver cranio-caudal diameter ≤16 cm	69%	58%	0.203
Spleen diameter ≥16 cm	47%	53%	0.517
Nodular liver	86%	68%	**0.018**
Caudate lobe hypertrophy	55%	64%	0.308
Paraesophageal varices	67%	63%	0.608
Splenorenal varices	72%	83%	0.166
Umbilical vein recanalization	60%	58%	0.765
Presence of ascites	55%	49%	0.515
Portal vein diameter ≤12 mm	58%	28%	**0.001**
**Duplex ultrasound findings**			
Portal vein flow velocity <16 cm/s	26%	20%	0.475
Hepatofugal flow	16%	7%	0.132
Hepatic artery resistive index ≥0.75	33%	39%	0.527

^a^Centrally acting medications include antidepressants, benzodiazepines, sleep aids, and antipsychotics.

^b^Diuretics include loop diuretics, thiazides, and spironolactone.

### Imaging studies

CT scan of the abdomen revealed that patients in the HE group had nodular liver counters and had smaller liver antero-posterior diameters (*P =* 0.018 and *P =* 0.001, respectively). Spleen dimensions and the presence of paraesophageal shunts, splenorenal shunts, umbilical vein recanalization, caudate lobe hypertrophy, liver cranio-caudal diameter did not have any association with the presence or absence of encephalopathy. Liver duplex ultrasound studies did not demonstrate any association between portal venous flow velocities, direction of flow or hepatic artery resistive indices with a patient's presentation (Table 2).

**Figure 2.**
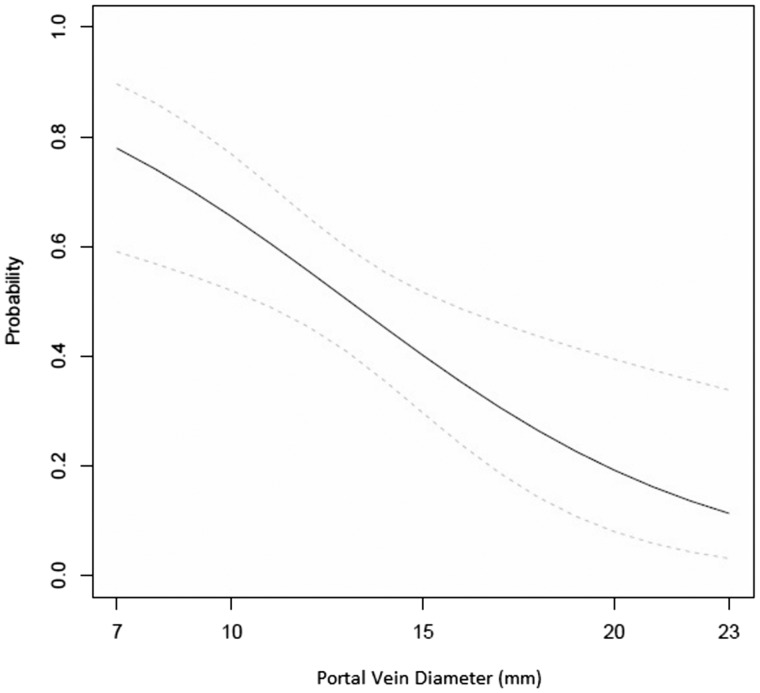
Estimated probability of encephalopathy (solid black line) and 95% confidence interval (dashed gray lines) from logistical regression model.

Mean portal vein diameter was 12.1 ± 2.9 mm in the HE group, as compared with 14.0 ± 3.1 mm in the control group (*P =* 0.001). The association between smaller portal vein size and HE was most pronounced when a portal vein diameter of 12 mm was used as a cut-off value (Figure 2). Two patients in the control group and 1 patient in the HE group had portal vein thrombosis and accurate assessment of portal vein size, velocity and flow direction were not possible.

### Multivariate analysis

Variables that were found to significantly correlate with HE were included in a multivariate logistical regression model. Portal vein diameter and the use of centrally acting medications and diuretics are significant predictors of encephalopathy in this multivariate model. None of the other factors was significant (Table 3).

**Table 3.** Estimated odds ratios (ORs) for multivariate logistic regression models

Variable	OR (95% CI)	*P*-value	c-index
Portal vein diameter, 1 mm decrease	1.18 (1.01–1.37)	0.035	0.76
Use of centrally acting medications	3.44 (1.28–9.27)	0.015	–
Use of diuretics	3.11 (1.08–8.98)	0.036	–
Liver antero-posterior diameter, 1 cm decrease	1.11 (0.91–1.37)	0.30	–
Nodular liver	2.11 (0.71–6.27)	0.18	–
MELD score, 1 point increase	1.03 (0.94–1.14)	0.48	–

CI = confidence interval; c-index = concordance-index; MELD = model for end-stage liver disease

When the data on the patients with larger portal vein diameter (>12 mm) was compared with patients with smaller portal vein diameter (≤12 mm), the average MELD in the smaller portal vein group was 13.8 ± 4.9, as compared with 12.9 ± 4.2 in the patients with the larger portal vein diameter (*P =* 0.29). Twenty-three percent of patients with smaller portal vein diameter had reversed portal flow, compared with just 3% in patients with larger portal vein (*P =* 0.002). Thirty-three percent of patients with smaller portal veins had a portal venous flow velocity of less than 16 cm/s, compared with 17% in the patients with larger portal vein diameter (*P =* 0.043). Although patients with smaller portal vein diameters were observed to have smaller liver and spleen dimensions and higher prevalence of shunts, the associations were not statistically significant. There was no significant difference in the use of beta blockers among patients with larger and smaller portal vein diameter (*P =* 0.21) (Table 4).

**Table 4.** Correlation between portal vein size and various radiographical parameters

Variable	PV ≤12	PV > 2	*P*-value
	*n =* 49	*n =* 65	
**CT scan findings**			
Liver antero-posterior diameter ≤16 cm	90%	74%	0.053
Liver cranio-caudal diameter ≤16 cm	65%	63%	0.8
Spleen diameter ≥16 cm	39%	55%	0.08
Nodular liver	75%	78%	0.71
Caudate lobe hypertrophy	59%	60%	0.93
Paraesophageal varices	71%	60%	0.2
Splenorenal varices	84%	74%	0.2
Umbilical vein recanalization	61%	60%	0.89
Presence of ascites	49%	55%	0.49
**Duplex ultrasound findings**			
Portal vein flow velocity <16 cm/s	33%	17%	**0.043**
Hepatofugal flow	23%	3%	**0.002**
Hepatic artery resistive index ≥0.75	39%	33%	0.51

## Discussion

The diameter and hemodynamics of the portal vein change with the advancement of cirrhosis [11]. The diameter of the portal vein is a reflection of the degree of the resistance it faces in the liver and the velocity of bloodflow within the portal vein. A normal portal vein diameter is considered to be around 10 mm [12]. Studies have shown that this value increases with the advancement of liver fibrosis and subsequently with the development of cirrhosis, where the mean portal vein diameter is reported to be around 14 mm [13–15]. A smaller portal vein was noted in patients with liver cirrhosis, who had a hepatofugal or reversed flow, those with a slower flow and in those patients with large collateral vessels [16–20]. Large collateral shunts, hepatofugal flow and slower flow were all found to be associated with an increased risk of encephalopathy [17, 18, 21].

Some of these correlations were confirmed in our analysis. Patients with smaller portal veins had slower portal vein flow, higher incidence of hepatofugal flow, higher incidence of HE and a higher prevalence of portosystemic shunts (the latter not being statistically significant). The smaller portal vein diameter observed in our study is most probably a reflection of the hemodynamic changes that occur with the advancement of cirrhosis and portal hypertension. As the resistance within the liver increases, the portal pressure and consequently the portal vein diameter will increase. With the increase in portal pressure, portosystemic shunts form, diverting blood away from the portal circulation to the systemic circulation. With time, the decrease in blood flow in the portal vein may be reflected in a smaller portal vein. With the advancement of cirrhosis, the liver’s ability to detoxify blood decreases and as a result patients will have higher incidence of HE. Changes in portal hemodynamics and the development of HE are both manifestations of advancing liver disease; the correlation between these two entities was noted in our study. We hypothesize that monitoring the diameter and hemodynamics of the portal vein over time may offer non-invasive clues that can point to the advance of liver disease and possibly predict the onset of complications related to this, such as HE. This correlation would need validation in prospective trials.

The limitations of this study include its retrospective nature, small sample size and the use of clinical parameters for diagnosis of encephalopathy. It is possible that some of the patients in the control group had minimal encephalopathy that could only be diagnosed with psychometric testing. Quantification of the degree of shunting was not possible with the current study design. It was also noted that a higher proportion of patients in the HE group were on diuretics and centrally acting medications (antidepressants, benozodiazipines, hypnotics and antipsychotics). Whether the HE caused these patients to have sleep disturbances, depressive or psychological symptoms or the medications themselves caused the HE symptoms could not be assessed by our study design. With regard to the use of diuretics, dehydration and electrolyte disturbances are known to exacerbate HE. The effect that the use of diuretics had on the volume status of these patients could not be assessed by our study design.

In conclusion, smaller portal vein diameter and the use of certain medications correlated with the presence of HE. In patients with liver cirrhosis, monitoring the portal vein diameter over time may identify patients whose disease has progressed and who are at risk of HE. A decrease in portal vein diameter over time may warrant screening for HE by more advanced psychometric testing, in addition to more aggressive control of constipation and other factors that may precipitate encephalopathy.

*Conflict of interest statement*: none declared.
